# Patterns of anopheline feeding/resting behaviour and *Plasmodium* infections in North Cameroon, 2011–2014: implications for malaria control

**DOI:** 10.1186/s13071-019-3552-2

**Published:** 2019-06-13

**Authors:** Wolfgang Eyisap Ekoko, Parfait Awono-Ambene, Jude Bigoga, Stanislas Mandeng, Michael Piameu, Narcisse Nvondo, Jean-Claude Toto, Philippe Nwane, Salomon Patchoke, Lili Ranaise Mbakop, Jerome Achille Binyang, Martin Donelly, Immo Kleinschmidt, Tessa Knox, Arthur Mbida Mbida, Alain Dongmo, Etienne Fondjo, Abraham Mnzava, Josiane Etang

**Affiliations:** 1 Institut de Recherche de Yaoundé (IRY), Organisation de Coordination pour la lutte Contre les Endémies en Afrique Centrale (OCEAC), P.O. Box 288, Yaoundé, Cameroon; 2 Laboratory of Animal Biology and Physiology, University of Douala, P.O Box 24157, Douala, Cameroon; 3 National Reference Unit (NRU) for Vector Control, The Biotechnology Center, University of Yaounde I, P.O. Box 3851, Messa, Yaoundé, Cameroon; 4 Faculty of Sciences, University of Yaounde I, P.O Box 337, Yaoundé, Cameroon; 5 Ecole des Sciences de la Santé, Université Catholique d’Afrique Centrale, B.P. 1110 Yaoundé, Cameroun; 6 National Malaria Control Programme, Ministry of Public Health, PO Box 14386, Yaoundé, Cameroon; 7 Department of Vector Biology, Liverpool School of Tropical Medicine, Pembroke Place, Liverpool, L3 5QA UK; 8 MRC Tropical Epidemiology Group, Department of Infectious Disease Epidemiology, London School of Hygiene and Tropical Medicine, Keppel Street, London, WC1E 7HT UK; 9 School of Pathology, Faculty of Health Sciences, University of the Witwatersrand, Johannesburg, South Africa; 10 Global Malaria Programme, World Health Organization, Avenue Appia 20, 1211 Geneva, Switzerland; 11 African Leaders Malaria Alliance, 3 Barack Obama Avenue, Dar es Salaam, Tanzania; 12 Faculty of Medicine and Pharmaceutical Sciences, University of Douala, P.O. Box 2701, Douala, Cameroon; 13 Institute for Insect Biotechnology, Justus-Liebig-University Gießen, Winchester Str. 2, 35394 Giessen, Germany

**Keywords:** Mosquitoes, Alternative hosts, Long-lasting insecticidal nets, Vector behaviour, Malaria infections, North Cameroon

## Abstract

**Background:**

Effective malaria control relies on evidence-based interventions. Anopheline behaviour and *Plasmodium* infections were investigated in North Cameroon, following long-lasting insecticidal net (LLIN) distribution in 2010.

**Methods:**

During four consecutive years from 2011 to 2014, adult mosquitoes were collected indoors, outdoors and in exit traps across 38 locations in the Garoua, Pitoa and Mayo-Oulo health districts. Anophelines were morphologically and molecularly identified, then analysed for blood meal origins and *Plasmodium falciparum* circumsporozoite protein (*Pf*-CSP). Blood from children under 5 years-old using LLINs was examined for *Plasmodium* infections.

**Results:**

Overall, 9376 anophelines belonging to 14 species/sibling species were recorded. *Anopheles gambiae* (*s.l.*) [*An. arabiensis* (73.3%), *An. coluzzii* (17.6%) and *An. gambiae* (*s.s.*) (9.1%)] was predominant (72%), followed by *An. funestus* (*s.l.*) (20.5%) and *An. rufipes* (6.5%). The recorded blood meals were mainly from humans (28%), cattle (15.6%) and sheep (11.6%) or mixed (45%). *Pf*-CSP rates were higher indoors (3.2–5.4%) *versus* outdoors (0.8–2.0%), and increased yearly (*χ*^2^ < 18, *df* = 10, *P* < 0.03). Malaria prevalence in children under 5 years-old, in households using LLINs was 30% (924/3088).

**Conclusions:**

The present study revealed the variability of malaria vector resting and feeding behaviour, and the persistence of *Plasmodium* infections regardless the use of LLINs. Supplementary interventions to LLINs are therefore needed to sustain malaria prevention in North Cameroon.

## Background

Unprecedented progress has been recorded in malaria control, especially in Africa where a 42% reduction in case incidence and a 66% decline in the mortality rate occurred between 2000 and 2016 [1]. However, 200 million cases were recorded in the African Region in 2017, accounting for 92% of the total case estimates in the world [1].

Approximately 150 different species of *Anopheles* mosquito have been described in sub-Saharan Africa (SSA); around 20 are primary or secondary malaria vectors [2]. The exceptionally high malaria transmission rates in SSA are in large part ascribed to the constant presence of efficient and competent vectors, especially those belonging to the *Anopheles gambiae* complex and the *An. funestus* group. The key elements that make these species highly efficient malaria vectors are anthropophagic and anthropophilic behaviour, i.e. a preference for humans as a source of blood, combined with indoor resting habits (endophily), and exploitation of breeding habitats created by human activities [3–5]. Knowledge of these vector innate feeding preferences and resting habits when combined with data on host availability/accessibility accurately predicts the intensity of malaria transmission [6]. Therefore, understanding how this propensity of malaria mosquitoes to feed on and live amongst humans, changes in response to anti-vector interventions is important for sustaining vector control. For instance, in South Africa, insecticide residual spraying (IRS) in houses was reported to significantly reduce the proportion of *An. arabiensis* that fed indoors on humans [7]. In this case, the IRS serves to decrease host availability and may either favour alternative innate host preferences in sections of the vector population or induce a “phenotypic plasticity” defined as the modification of host selection without changes in innate (genetic) host preference [8]. In the absence of insecticide resistance, the lack of host-selection phenotypic plasticity in human biting species may cause them to enter the houses and thus increases their likelihood of being killed. Conversely, mosquitoes that do not enter houses will have a selective survival advantage and the intervention may have little effect, especially if these mosquitoes are opportunistic, resistant to insecticides or exhibit plasticity in host selection.

The major malaria vectors in Africa exhibit differential abilities to adapt in widely varying environmental conditions, enabling their survival [4]. For instance, *An. gambiae* is known to exhibit a high preference for human hosts whereas *An. arabiensis* is regarded as more opportunistic [9–12]. However, both species, as well as their sibling species *An. coluzzii* and species of the *An. funestus* group, have been reported to rapidly develop pyrethroid resistance [13], which renders malaria vector control very complex. To design better vector control and disease prevention measures, it is essential to characterize the behavioural patterns of vector populations over the time and in a range of environmental conditions, especially in the context of pyrethroid resistance among sibling taxonomic units. Such approach would probably be more appropriate than tailoring the interventions according to data on the whole group or the whole complex of vector species.

In Cameroon, the most efficient malaria vector species belong to the *An. gambiae* complex [*An. gambiae* (s.s.), *An. coluzzii* and *An. arabiensis*], followed by *An. funestus*, *An. nili* and *An. moucheti* groups. Species such as *An. paludis*, *An. pharoensis*, *An. hankocki* and *An. rufipes* play secondary roles in malaria transmission [14–20]. Furthermore, pyrethroid resistance is widespread in the species of the *An. gambiae* complex [21–25] and deltamethrin resistance was more recently reported in *An. rufipes* from the North Region [26]. The number of malaria cases in Cameroon was estimated at 1.2 million in 2013, the majority of which were due to *Plasmodium falciparum* [27]. Efforts to curb malaria based on widespread use of long-lasting insecticidal nets (LLINs) alongside intensification of case management led to a significant reduction of the prevalence in the general population from 46.3% in 2008 to 17.6% in 2017 [27, 28]. Insecticide treated nets (ITNs) act as physical and chemical barriers, preventing access by vector mosquitoes to human hosts, and reducing subsequent blood-feeding and *Plasmodium* parasite transmission. In order to inform vector control strategies and to identify any factors that may compromise the impact of the interventions, there is a need for a close monitoring of vector populations and parasite transmission. Key entomological indicators to monitor include vector abundance, resting/feeding behaviour and susceptibility to the insecticides used in ITNs or IRS. However in Cameroon, while the status of malaria vector resistance is well known, to date, data on their resting and feeding behaviour in the context of wide LLIN use is scarce. The present report presents data from four cross sectional surveys on malaria vector behaviour conducted yearly from 2011 to 2014 and a cross sectional survey on *Plasmodium* infections in North Cameroon (2013). This study is part of a multi-country project to assess the impact of insecticide resistance on the effectiveness of LLINs or IRS interventions [29].

## Methods

### Study sites and environmental landscapes

The study was conducted in 38 clusters (defined as villages or groups of hamlets with no less than 500 houses) distributed across 3 health districts (HD), namely Garoua, (9°30′N, 13°40′E), Pitoa (9°21′N, 13°31′E) and Mayo Oulo (9°46′N, 13°44′E).

The three study HDs lie within the Soudanian climate domain with 700–1000 mm of annual rainfall with 3 months of rains (July to October) and 9 months of dry season (November to June). The average temperature in this region is 35 ± 5 °C. The region contains 1,227,000 inhabitants and makes up 65,576 km^2^, with 12–25 people per km^2^ in Mayo Oulo, 25–50 people per km^2^ in Pitoa and 50–100 people per km^2^ in Garoua. The region is strewn with rivers and the river valleys are adequate rice growing areas thus they constitute excellent breeding sites for mosquitoes. *Anopheles gambiae* (*s.l*.) and *An. funestus* group are the main malaria vectors in this area.

### Mosquito collection

The study began in November 2011, 18 months after the launch of a LLIN mass distribution campaign in North Cameroon in June 2010. Yearly cross-sectional entomological surveys were conducted during the high transmission seasons between September and November for four consecutive years from 2011 to 2014. To monitor the effect of LLINs on local mosquito populations, only houses that possessed LLINs were selected for mosquito collection, and the same houses were visited each year. Adult mosquitoes were collected across 380 (38 × 10) houses randomly chosen in 38 study clusters, using 3 conventional sampling methods: window exit traps (WETs), outdoor clay-pots (OCPs) as outdoor shelters and pyrethrum spray catches (PSCs) [30].

Ten WETs (one per sleeping bedroom window) were set up on windows between 5:00 and 6:00 pm and then checked the next day between 07:00 and 09:00 am for 2 consecutive nights.

For outdoor collection, three 25–30 l OCPs containing 5–10 litres of water were placed in each of 3 selected compounds among those used for WETs (i.e. 9 pots per cluster). The pots were placed at 6:00 pm, preferably at the back of the house close to a bedroom and away from areas with a lot of human activities to avoid disturbing resting mosquitoes. Mosquitoes resting in the pots were collected the next day between 7:00 and 9:00 am for two successive days using mouth aspirators.

PSCs were performed between 6:00 and 9:00 am in all rooms of the 380 study houses once the WET and OCP collections were completed. White sheets were laid on the floor and over the furniture. Then all windows and doors were shut and rooms were sprayed with pyrethrum or pyrethroid based aerosols. The houses were then closed for 10–15 min to knock down the mosquitoes resting indoors. Mosquitoes that fell on the sheets were collected using forceps.

Mosquito samples from the three collection methods were kept separately for subsequent analysis.

### Mosquito processing

Field collected mosquitoes were morphologically identified using keys for the species of the genus *Anopheles* [31, 32] and classified as fed or unfed, based on abdominal appearance. Each mosquito was then dissected and body parts placed into 3 tubes (head/thorax, abdomen and legs/wings). DNA was extracted from the legs and wings of *An. gambiae* (*s.l.*) specimens with CTAB 2% [33] and used for species identification by means of a PCR-RFLP method [34]. All heads/thoraxes of the *Anopheles* samples were screened for *Plasmodium falciparum* circumsporozoite protein (CSP) and abdomens of fed specimens checked for blood meal origins using ELISA [35, 36]. Monoclonal antibodies against human, cattle, pig, horse, chicken and sheep blood were used for blood meal ELISA.

### Malaria prevalence survey

A malaria prevalence cross-sectional survey was conducted in each cluster of the three study HDs in October 2013. Data on LLINs ownership and usage were collected and 40–45 households owning LLINs were sampled per cluster for *Plasmodium* parasite screening in children 0.5–5 years-old. Children were asked if they had slept under bed nets the previous night and if yes, whether they used the nets regularly or not. Furthermore, from the beginning of the study in 2011 and during the study period, the parents were encouraged by the study team to ensure net usage by their children, and the process was monitored by the community health workers. After informed consent of the parents, malaria diagnosis by rapid diagnostic test (RDT) (SD BIOLINE Malaria Ag Pf/Pan^(r)^ 05FK60; Standard Diagnostics Inc., Suwon City, South Korea) was performed by health personnel on the children, following the manufacturer’s instructions. Children positive by RDT were treated with artesunate-amodiaquine according to the national guidelines. Blood slides and thick blood films were also taken. Microscopy was conducted using 10% Giemsa stain, and examined after completion of fieldwork by laboratory technicians. A second reading was carried out by a technician at OCEAC Laboratory (Yaoundé, Cameroon) and results were compared; the discrepancies were double checked. All technicians were blinded for RDT results.

### Statistical analysis

The human blood index (HBI) was calculated by summing species blood meals from human only and mixed blood meals between human and animals divided by the total blood meals registered per species. Sporozoite rates were calculated for each species, as the proportion of mosquitoes tested positive for ELISA CSP. The Pearson Chi-square test was performed to compare LLIN ownership and use in the three HDs, as well as sporozoite rates between malaria vector species, collection methods and years of collection. A one-way ANOVA was conducted to compare the overall vector densities between indoors, exit and outdoor collection, as well as human blood indices. The Anderson-Darling 3-sample test of null was used to compare average relative densities of the major malaria vectors across the three HDs.

## Results

### Malaria vector species and abundance

**Fig. 1:**
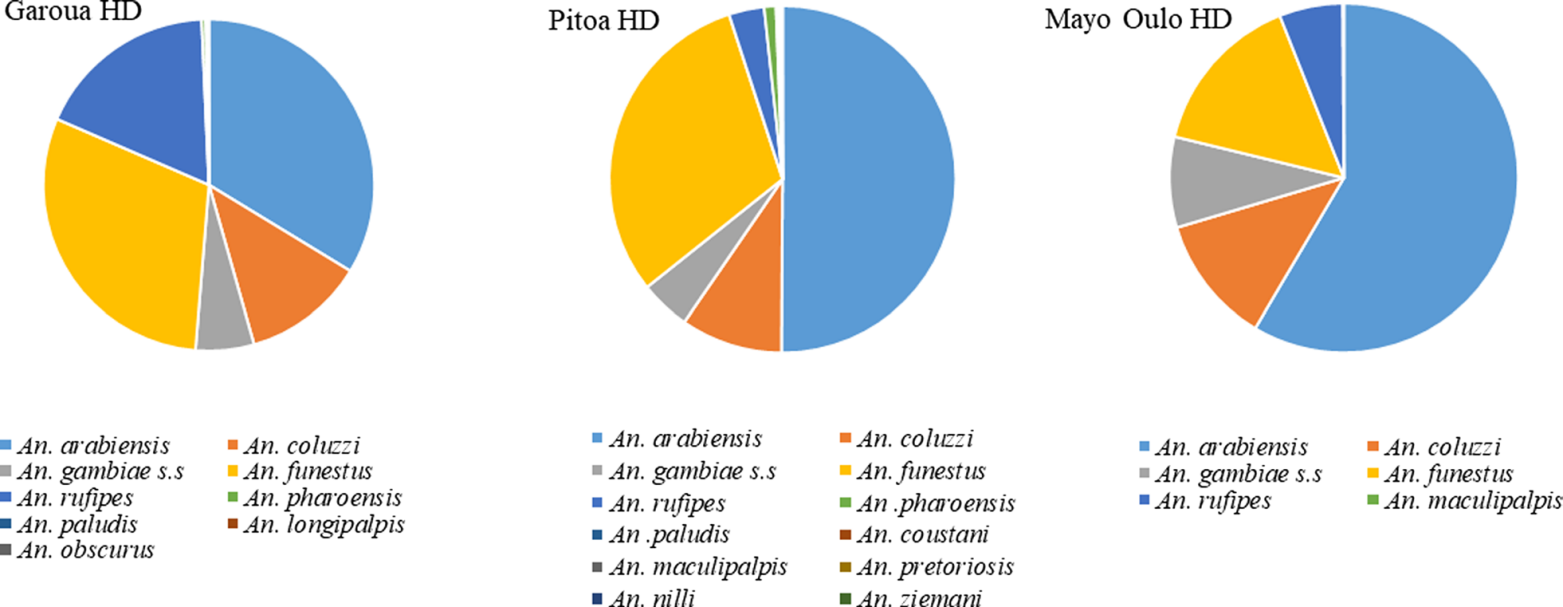
Species composition of anopheline samples collected in the Garoua, Pitoa and Mayo Oulo Health Districts (HD) from 2011 to 2014

Data on anopheline diversity and abundance are presented in Fig. 1. A total of 9376 anopheline specimens belonging to 14 species were collected across the 38 study clusters. The anopheline fauna was more diverse in the Pitoa HD with 12 species recorded, followed by the Garoua HD with 9 species recorded, while only 6 species were identified in samples from the Mayo Oulo HD. *An. gambiae* (*s.l.*) was predominant (72.0%, *n* = 6758), followed by *An. funestus* (*s.l.*) (20.5%, *n* = 1922) and *An. rufipes* (6.5%, *n* = 614). All other species represented less than 1% of the total (*n* = 82).

Among the 6758 *An. gambiae* (*s.l.*) collected, 3968 specimens randomly selected for molecular identification revealed three sibling species: *An. arabiensis* (73.3%), *An. coluzzii* (17.6%) and *An. gambiae* (9.1%). While *An. coluzzii* and *An. gambiae* were at relatively low proportion across the three HDs (5–12%), *An. arabiensis* was the predominant species of the entire fauna from the Mayo Oulo and Pitoa HDs (50–58%), followed by *An. funestus* (15–31%). In the Garoua HD, *An. arabiensis* occurred at similar proportions with *An. funestus* (30–34%), followed by *An. rufipes* (18%).

### Long-lasting insecticidal net usage and anopheline density indoors and outdoors

The proportions of households owning at least one LLIN varied between 60–71%, and the rates of LLINs utilization between 43–54%, with no significant difference from one HD to another (*χ*^2^ < 3.38, *df* = 1, *P* > 0.1).

A total of 2592 clay pots and 2880 exit traps were used for collection of outdoor and exiting mosquitoes, while 1440 rooms were sprayed for indoor collection; the mean relative vector densities are summarized in Table 1. In the Pitoa HD, the relative anopheline density (mean ± SD) was higher indoors (3.75 ± 0.96 anophelines/room), compared with exit traps and outdoors (1.47 ± 1.26 and 2.19 ± 1.02 anophelines/trap, respectively), although the difference was not statistically significant (*F*_(2,46)_ = 4 .00, *P* = 0.05). However, in the Garoua and Mayo Oulo HDs, there were no significant differences between mean relative densities among the different collection methods (*F*_(24,70)_ < 1.67, *P * > 0.2).

**Table 1:** Long-lasting insecticidal net ownership and usage *versus* anopheline densities indoors, in exit traps and outdoors across the Garoua, Pitoa and Mayo Oulo health districts

Health district	LLINs ownership (%)	LLINs use (%)	Collection method	No. of traps	No. of *Anopheles* caught	Mean no. ± SD of *Anopheles*/trap	*F-*value	*P*-value
Garoua	60.1	42.9	Outdoors	1080	1307	1.21 ± 1.27	1.67	0.24
			Exit trap	1200	1158	0.97 ± 0.70		
			Indoors	600	1091	1.82 ± 0.74		
Pitoa	68.3	54.5	Outdoors	864	1889	2.19 ± 1.02	4.00	0.05
			Exit trap	960	1412	1.47 ± 1.26		
			Indoors	480	1799	3.75 ± 0.96		
Mayo Oulo	70.8	53.8	Outdoors	648	172	0.27 ± 0.30	0.43	0.56
			Exit trap	720	225	0.31 ± 0.38		
			Indoors	360	325	0.90 ± 0.29		

*Abbreviations*: LLINs ownership (%), percentage of households owning long-lasting insecticidal nets; LLINs use (%), percentage of households using long-lasting insecticidal nets; SD, standard deviation; *F*-value (one-way ANOVA); *P*-value: significance at 0.05%

Regarding species distribution as shown in Table 2, there was no significant difference between the relative densities of *An. arabiensis*, *An. coluzzii* or *An. gambiae* (*s.s.*) caught outdoors, in exit traps or indoors (*F*_(26,48)_ < 1.83, *P* > 0.1). Conversely, *An. funestus* (*s.l.*) was mostly found outdoors compared with indoors and exit traps (*F*_(4,20)_ > 8.56, *P* < 0.01), suggesting exophilic tendencies. The same tendency was observed in *An. rufipes*, although the differences were not statically significant.

**Table 2:** Mean relative densities of major malaria vector species per collection method

Species	ADE ± SD	ADI ± SD	ADO ± SD	*P*-value
*An. arabiensis*	0.55 ± 0.57	0.92 ± 0.72	0.66 ± 0.54	0.2090
*An. coluzzii*	0.12 ± 0.13	0.22 ± 0.18	0.16 ± 0.14	0.1426
*An. gambiae* (*s.s*.)	0.05 ± 0.06	0.12 ± 0.11	0.09 ± 0.10	0.1980
*An. funestus*	0.14 ± 0.16	0.34 ± 0.39	0.91 ± 0.83	0.0034
*An. rufipes*	0.18 ± 0.19	0.11 ± 0.08	0.34 ± 0.25	0.0781

*Abbreviations*: ADE, average density in exit trap; ADI, average density indoors; ADO, average density outdoors; SD, standard deviation

### Temporal variations of vector densities

**Fig. 2:**
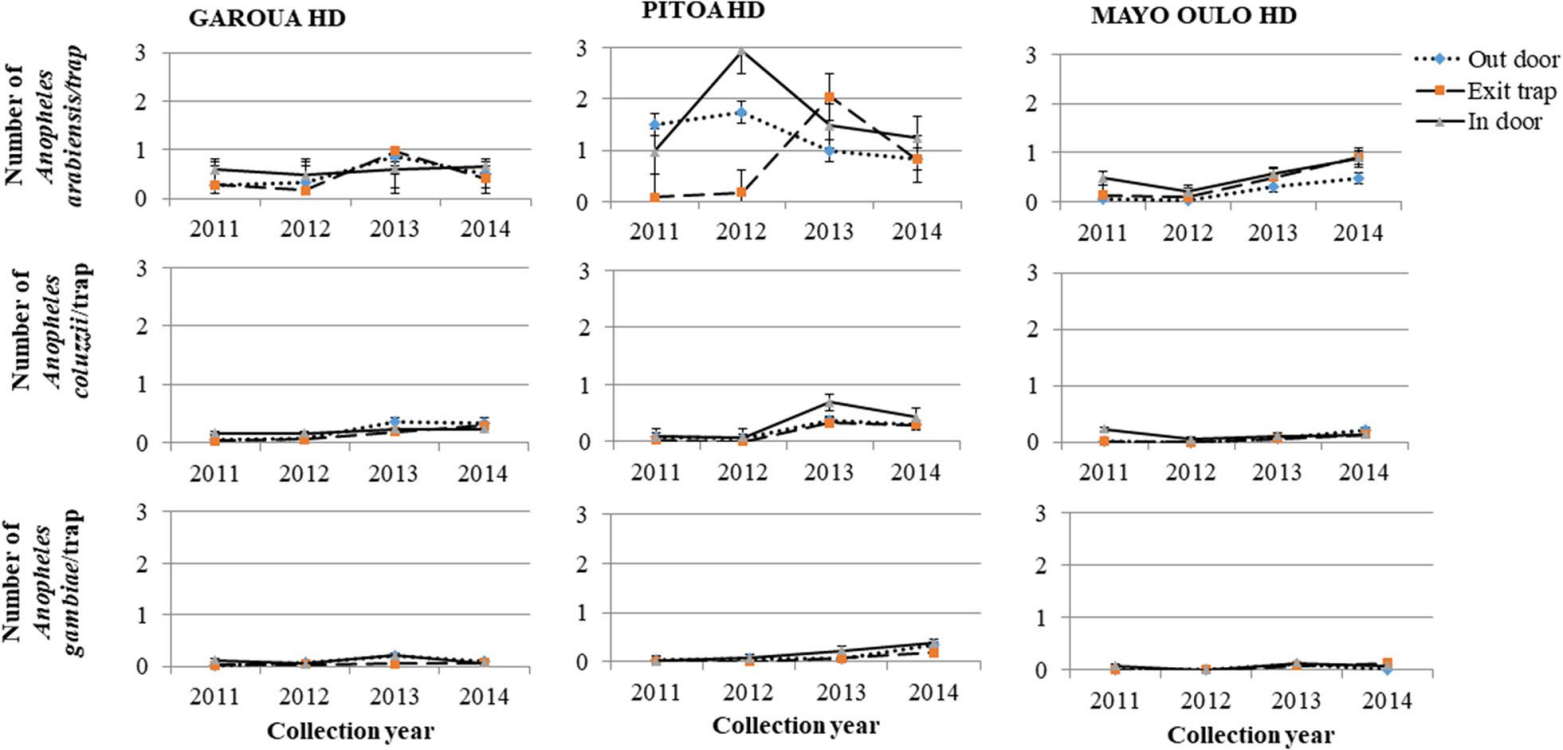
Temporal variations of the densities of *Anopheles arabiensis*, *Anopheles coluzzii* and *Anopheles gambiae* collected outdoors, in exit traps and indoors

**Fig. 3:**
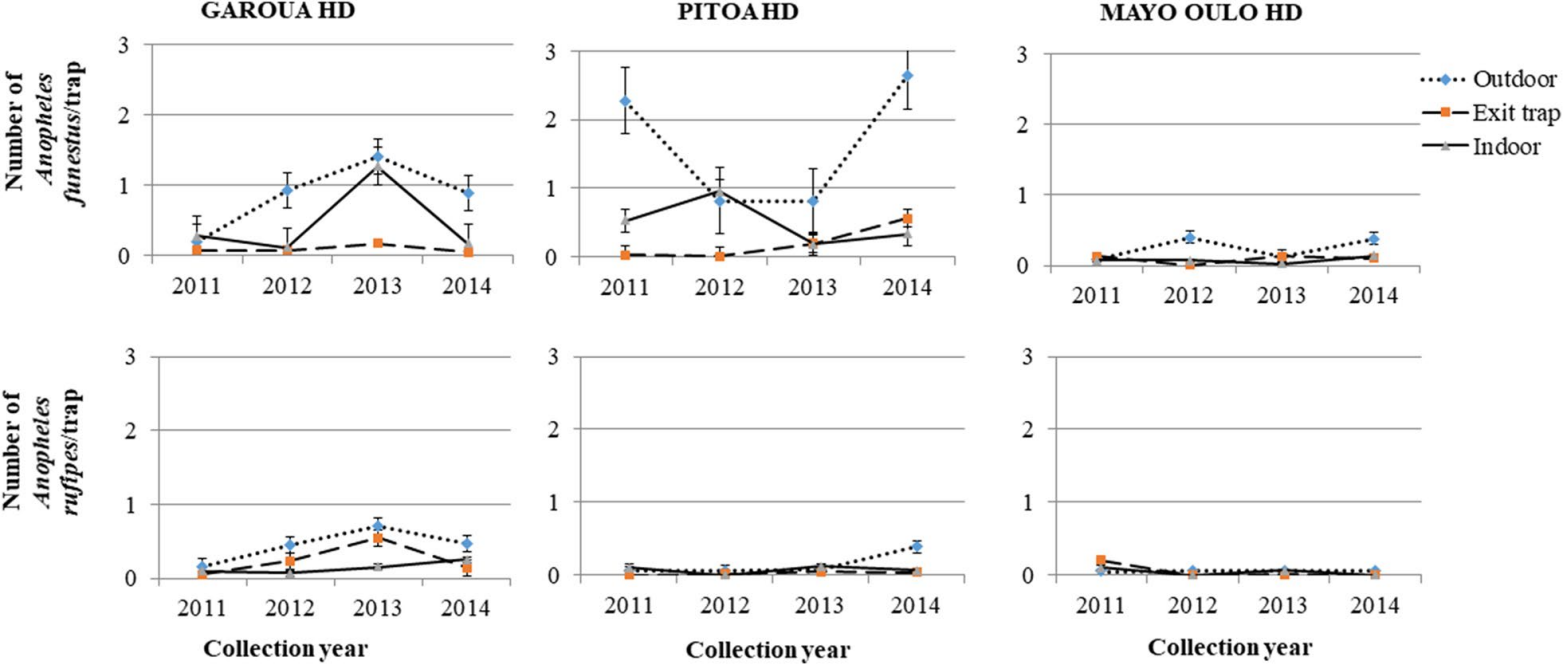
Temporal variations of the densities of *Anopheles funestus* and *Anopheles rufipes* collected outdoors, in exit traps and indoors

Densities of *An. arabiensis*, *An. coluzzii*, *An. gambiae* (*s.s.*) generally increased over the years, either indoors, in exit traps or outdoors in some cases (*F*_(3,6)_ > 8.94, *P * < 0.05). With *An. arabiensis* from Pitoa, *An. coluzzii* from Mayo Oulo and *An. gambiae* from Garoua, the increase was not linear (*F*_(16,37)_ < 2.17, *P * > 0.1) (Figs. 2, 3). However, no significant temporal variations of vector densities were observed outdoors *versus* indoors and exit traps, except in Pitoa where *An. arabiensis*, which was mostly caught indoors than outdoors and exit traps in 2012, was equally caught by the three collection methods in 2014.

*Anopheles funestus* and *An. rufipes* were mostly collected outdoors over the course of the study, and no significant variations were recorded in their densities during the four years of the survey (*F*_(9,80)_ < 2.77, *P * > 0.1).

### Blood-feeding indices

A total of 3014 blood-fed *Anopheles* corresponding to 32% of the collected samples were analysed for blood meal origins. Data summarized in Table 3 revealed variable proportions of single-host blood meals and mixed blood meals from one vector species to another and from one health district to another. However, the overall proportion of single-host blood meals (53%, *n* = 1603) was significantly higher than mixed blood meals (47%, *n* = 1411) (*χ*^2^ = 24.46, *df* = 8, *P* < 0.005). Single-host blood meals were obtained mainly from humans (28%, *n* = 837), cattle (15.6%, *n* = 470) and sheep (11.6%, *n* = 350). Mixed blood meals were from human/animal (mainly between human/cattle and human/sheep) (24%, *n* = 724) and from animal/animal (mainly between cattle/sheep/pig) (23%, *n* = 687). Blood from humans, cattle, sheep and pigs was recorded in the five major malaria vector species, whereas blood from fowl was found only in *An. colluzzi*, *An. gambiae* and *An. funestus*.

**Table 3:** Trophic preference, human and animal blood indices of the five major malaria vectors

Health district	Species	Hosts (%)							HBI
		Humans	Cattle	Sheep	Pigs	Fowl	Mixed H/A	Mixed A/A	
Garoua	*An. arabiensis* (*n* = 386)	37.8	11.7	7.0	3.9	0.5	25.4	13.7	**0.63**
	*An. coluzzii* (*n* = 159)	44.7	6.9	3.8	3.8	1.8	28.3	10.7	**0.73**
	*An. gambiae* (*s.s.*) (*n* = 73)	61.4	5.5	5.5	2.9	0.0	11.0	13.7	**0.73**
	*An. funestus* (*n* = 540)	12.2	18.9	27.0	0.6	0.2	13.3	27.8	0.26
	*An. rufipes* (*n* = 266)	19.5	15.0	13.5	4.3	0.0	12.0	35.7	0.32
	Total (*n* = 1424)	26.7	14.2	15.4	2.6	0.4	17.9	22.8	0.45
Pitoa	*An. arabiensis* (*n* = 665)	29.9	14.1	10.8	1.9	0.0	23.0	20.3	**0.53**
	*An. coluzzii* (*n* = 113)	39.8	8.0	2.7	0.9	0.0	39.8	8.8	**0.80**
	*An. gambiae* (*s.s.*) (*n* = 57)	52.6	12.3	1.6	0.0	0.0	24.7	8.8	**0.77**
	*An. funestus* (*n* = 357)	11.8	7.8	9.2	1.5	0.0	35.0	34.7	0.47
	*An. rufipes* (*n* = 48)	10.4	12.5	4.2	4.2	0.0	35.4	33.3	0.46
	Total (*n* = 1240)	25.9	11.6	9.0	1.6	0.0	28.5	23.4	**0.54**
Mayo Oulo	*An. arabiensis* (*n* = 194)	42.3	7.2	1.0	0.0	0.0	33	16.5	**0.75**
	*An. coluzzii* (*n* = 51)	51.0	0.0	0.0	0.0	0.0	41.2	7.8	**0.92**
	*An. gambiae* (*s.s.*) (*n* = 30)	60.0	0.0	0.0	0.0	0.0	33.3	6.7	**0.93**
	*An. funestus* (*n* = 56)	12.5	1.75	5.4	1.75	0.0	28.6	50	0.41
	*An. rufipes* (*n* = 19)	15.8	21.1	10.5	0.0	0.0	21.1	31.5	0.37
	Total (*n* = 350)	15.8	21.1	10.5	0.0	0.0	32.9	20.6	**0.72**

*Abbreviations*: *n*, number of blood meals tested; Mixed H/A, mixed human-animal blood; Mixed A/A, mixed animal-animal blood; HBI, human blood index

*Note*: Numbers in bold refer to HBI higher than 0.50

The three sibling species of the *An. gambiae* complex showed 30–61% human blood meals, 11–41% mixed human/animal and 7–20% mixed animal/animal meals. The resulting HBIs ranged from 0.53 to 0.93 and were not statistically significant between the three species (*F*_(12,16)_ = 2.89, *P* = 0.13). Conversely, 10–20% blood meals of *An. funestus* and *An. rufipes* were from human, 1–21% from animals, 12–35% from human/animal and 6–50% from animal/animal. Their HBIs ranged from 0.26 to 0.54 and were significantly lower than those of the three sibling species of the *An. gambiae* complex (*F*_(2,120)_ = 34.37, *P* < 0.005).

No significant differences were observed when comparing the mean HBIs of the 5 vector species between the 3 HD (*F*_(20,120)_ < 1.57, *P* > 0.62).

### *Plasmodium falciparum* circumsporozoite rates

Overall, 111 specimens among the 5153 analysed tested positive to *P. falciparum* CSP protein, with a mean sporozoite rate of 2.15%. The infected specimens belonged to 7 anopheline species out of the 14 species recorded in the study sites. The three sibling species of the *Anopheles gambiae* complex had the highest infection rates, corresponding to 4.76% (14/294), 3.95% (24/607) and 2.28% (59/2586) for *An. gambiae* (*s.s.*), *An. coluzzii* and *An. arabiensis*, respectively. The infection rates were higher in *An. gambiae* and *An. coluzzii*, compared with *An. arabiensis* (*χ*^2^*=* 9.74, *df* = 2, *P* = 0.007). For *An. funestus* and *An. rufipes*, the infection rates were less than 1%, i.e. 0.77% (10/1403) and 0.71% (2/258), respectively. For *An. pharoensis* and *An. longipalpis*, 1/10 and 1/1 analysed specimens were CSP positive, respectively.

The distribution of infection rates among mosquito samples collected indoors, outdoors and exit traps across years of mosquito collection is summarized in Table 4. In the Garoua and Pitoa HDs, the infection rates were higher in anopheline samples collected indoors (3.22–3.50%) compared with exit traps (1.62–2.08%) and outdoor samples (0.80–1.30%) (*χ*^2^ < 18, *df* = 10, *P* < 0.03). Furthermore in the Pitoa HD, the infection rates significantly increased between 2011 and 2014 from 0.50% (3/600) to 2.49% (25/1004) (*χ*^2^ = 16.56, *df* = 4, *P* = 0.0008); while in the Garoua HD, two peaks of infections were recorded in 2012 (2/47, 4.25%) and 2014 (25/540, 4.63%). In the Mayo Oulo HD, the infection rates remained between 2.13% (1/47) and 4.61% (6/130) across the 4-year study period (*χ*^2^ = 0.57, *df* = 3, *P* = 0.90) and the difference between indoor, exit and outdoor infectivity was not statistically significant (*χ*^2^ = 2.06, *df* = 5, *P* = 0.36)

**Table 4:** *Plasmodium falciparum* circumsporozoite protein (CSP+) rates in *Anopheles* samples by method and year of collection

HD	Collection method	Year of collection								Total		*χ* ^2^	*P*-value
		2011		2012		2013		2014					
		*n*	CSP_+_ (%)	*n*	CSP_+_ (%)	*n*	CSP_+_ (%)	*n*	CSP_+_ (%)	*n*	CSP_+_ (%)		
GRA	OUT	32	0	11	0	479	0.83	250	2.40	772	1.30	7.77	0.02
	EXIT	16	0	9	0	287	1.05	121	4.96	433	2.08		
	IN	66	3.03	24	8.33	370	1.35	169	7.69	629	3.50		
	Total	114	1.75	47	4.25	1137	1.06	540	4.63	1834	2.23	22.42	0.00005
PIT	OUT	407	0.25	184	0	181	1.10	484	1.45	1256	0.80	17.98	0.0001
	EXIT	14	0	20	0	293	1.02	227	2.64	554	1.62		
	IN	179	1.12	351	1.12	295	5.76	293	4.09	1118	3.22		
	Total	600	0.50	555	0.72	769	2.86	1004	2.49	2928	1.84	16.56	0.0008
MYO	OUT	3	0	21	0	30	0	44	4.55	98	2.04	2.06	0.36
	EXIT	10	0	1	0	43	4.65	72	2.78	126	3.17		
	IN	34	2.94	5	20.00	57	7.02	71	4.22	167	5.39		
	Total	47	2.13	27	3.70	130	4.61	187	3.74	391	3.82	0.57	0.90

*Abbreviations*: *n*, number of mosquitoes tested; HD, health district; GRA, Garoua; PIT, Pitoa; MYO, Mayo Oulo; OUT, outdoors; EXIT, exit trap; IN, indoors

### Prevalence of *Plasmodium* infections in children under 5 years-old

**Fig. 4:**
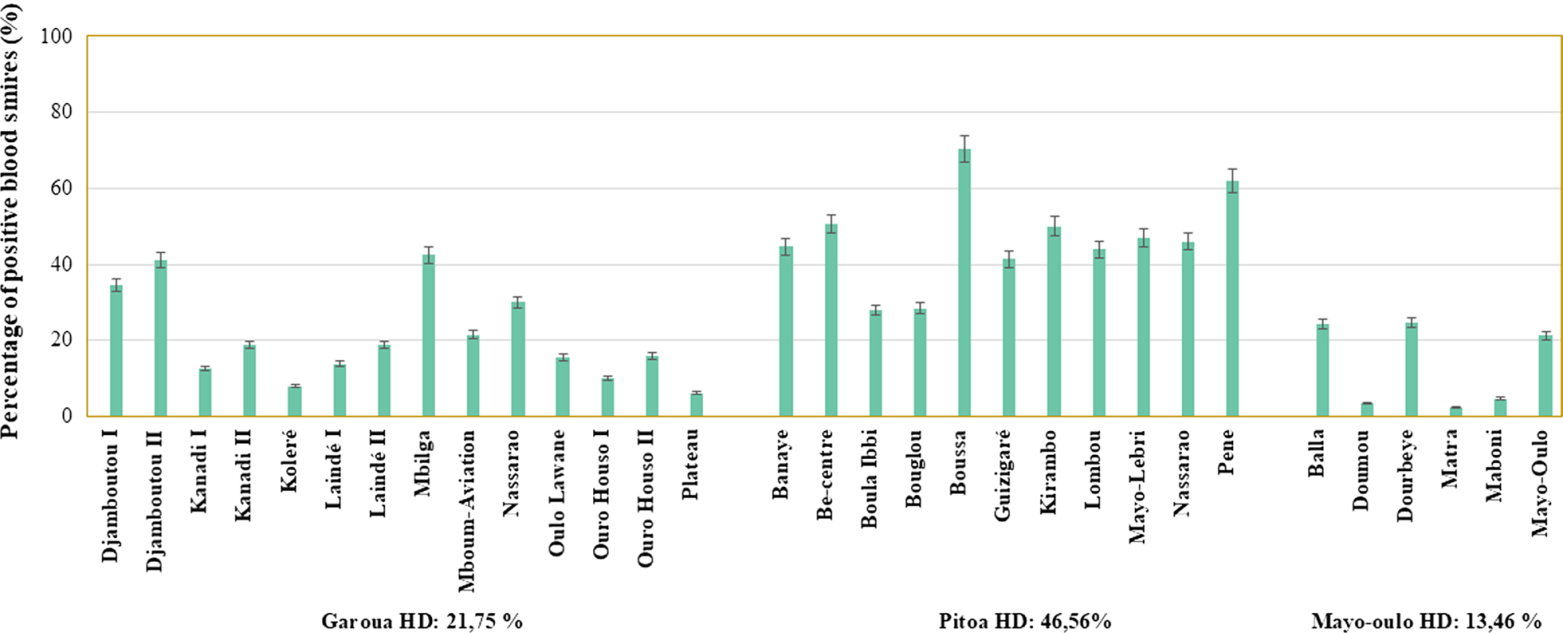
Malaria prevalence among children under 5 years-old from households using long-lasting insecticidal nets in the Garoua, Pitoa and Mayo Oulo health districts (HD)

Data on *Plasmodium* infections in children under 5 years-old were collected from 31 out of the 38 clusters with entomological data. A total of 3088 children (≈ 80 children from 30 households/cluster) were examined for malaria infection using RDT and confirmed using microscopy. The mean age at the recruitment was 27.9 (6–60) months. The number of fever cases (temperature ≥ 37.5 °C) was 1494/3088 (48.4%) and the number of positive RDT with fever was 958/1494 (64%). The overall malaria prevalence for the study population was 30% (924/3088), increasing from Mayo Oulo HD (16%, 85/540), to Garoua HD (23%, 340/1503) and Pitoa HD (48%, 499/1045) (Fig. 4). A high variability was seen in infection rates among the clusters of the same HD, i.e. 28–70%, 6–42% and 2–25% in the Pitoa, Garoua and Mayou HDs, respectively. The infections were solely due to *P. falciparum*.

## Discussion

Entomological and epidemiological monitoring and evaluation of interventions are of great importance for timely and effective response of malaria control programmes. A few studies have been carried out to describe the distribution of malaria vectors and their role in disease transmission in the northern savannah of Cameroon [15, 18–20]. However, little is known about the behavioural response of these vector populations to the wide use of LLINs, while evidence show that successful malaria elimination strategies require interventions that target changing vector behavior [37]. In this study, locally made clay pots were tested and used for the first time to monitor outdoor resting mosquitoes, alongside indoor spray catches for indoor collection and exit traps for collection of mosquitoes escaping from houses through the windows, in areas with 60–70% LLIN coverage. The used OCPs may have underestimated the relative densities of outdoor resting mosquitoes, because mosquitoes might seek alternative shelters (eaves of huts, canal water pipes, undersides of bridges, cracks and holes in the ground, granaries, etc.) [38–40]. However, the three collection methods yielded consistent samples of mosquitoes for subsequent analysis during a four years longitudinal survey.

Fourteen malaria vector species were identified in study HDs, with three sibling species of the *An. gambiae* complex, i.e. *An. arabiensis*, *An. coluzzii* and *An. gambiae* (*s.s.*) being the major vectors, followed by* An. funestus* (*s.l.*) and* An. rufipes*. Species of the *An. gambiae* complex displayed high HBIs, with increasing relative densities either indoors, across house openings or outdoors; while* An. funestus* (*s.l.*) and* An. rufipes* were mostly found outdoors, with lower HBIs. The predominance of *An. arabiensis* in the three study HDs located in semi-arid areas is consistent with previous observations from the same areas [18, 23, 24, 41] and elsewhere in Africa [42–45]. In the Umbugwe area (now called Magugu) of Northern Tanzania,* An. arabiensis* has shown a tendency to exit from houses after feeding, a behavioral pattern normally referred to as exophily [46]. Elsewhere in the Kisumu area, Kenya, Highton et al. [47] reported that *An. arabiensis* showed a tendency to occur outdoors 2.2 times more frequently than indoors, while Joshi et al. [48] reported 2.8 times. However, the present study found no significant difference between the proportions of *An. arabiensis* indoors, in exit traps and outdoors. More interestingly, *An. gambiae*, *An. coluzzii* and *An. rufipes* were also largely distributed across the three collection methods; the three species were previously reported to develop resistance to deltamethrin, the same as *An. arabiensis* in the three study HDs. The synchronous presence of the four species across the collection methods may be related to the heterogeneity of their populations in terms of frequencies of physiological resistance to deltamethrin. Etang et al. [49] previously reported a significant dependence of indoor relative densities of *An. arabiensis* and *An. coluzzii* on increasing deltamethrin resistance in the Garoua, Pitoa and Mayo Oulo HDs.

Whereas LLINs were expected to progressively reduce or even eliminate malaria vectors, with subsequent decline of infection rates, the infection rates were higher in mosquitoes collected indoors *versus* outdoors and increasing from one year to another, suggesting ongoing malaria transmission regardless of the use of LLINs. Moreover, *An. funestus* (*s.l.*) was found mostly exophilic and fed on animals. In Burkina Faso, a behavioural divergence was observed between highly anthropophagic and sympatric chromosomal forms of *An. funestus* known as Folonzo (mosly found indoors) and Kiribina (over-represented outdoors), suggesting that indoor interventions may be less effective against the Kiribina form [50]. Fontenille et al. [51] also found that although very few *An. funestus* collected indoors in the Manarintsoa area of Madagascar fed on animals, 65.0% of the exophilic population had fed on bovid blood. The exophilic and zoophilic propensities of *An. funestus* reported in this study may be related to species or chromosomal composition of sampled populations. Molecular analysis of *An. funestus* (*s.l.*) samples from Garoua collected in 2001–2002 revealed three species: *An. funestus* (*s.s.*), *An. leesoni* and *An. rivulorum*-like from larval collections, *versus* 93% *An. funestus* (*s.s.*) and 7% *An. leesoni* from indoor collections [52]. The biology and vectorial capacity of the three sibling species are highly contrasting. *Anopheles funestus* (*s.s.*) is endophilic and anthropophilic, and considered a major vector of human malaria, while *An. leesoni* and *An. rivulorum*-like are primarily zoophilic and exophilic, but can also transmit human malaria [2, 53]. Therefore, in the present study, indoor samples carrying human blood may belong to *An. funestus* (*s.s.*) species, while exophilic samples carrying animal blood may be of *An. leesoni* or *An. rivulorum*-like species. Alternatively, the exophily of *An. funestus* (*s.l.*) could also be related to excito-repellency and deterrence of LLINs, assuming that these species were susceptible to deltamethrin. Nevertheless, Menze et al. [54] recently reported multiple insecticide resistance in *An. funestus* (*s.s.*) from Gounougou, located 40 km south-east of Garoua on the right bank of the Benoue River. Further studies are needed to investigate insecticide resistance in the species of the *An. funestus* group from the Garoua, Pitoa and Mayo Oulo HDs.

Among the seven species found infected with the *P. falciparum* parasite, six had already been incriminated in the study HDs [15, 18–20]. The seventh species, *An. longipalpis*, for which the only specimen collected was found infected with *P. falciparum*, needs further investigations, since this species is primarily zoophilic [2] and no strong conclusions could be drawn due to its small sample size. Interestingly, the six major vector species fed on human and animals, mainly cattle and sheep, with a variety of mixed blood meals. *Anopheles funestus* and *An. rufipes* obtained a greater proportion of their blood meals from animals. This plasticity of the vector trophic habits may influence the epidemiology of malaria, leading to residual transmission after the main endophilic and endophagic vectors have been reduced by the interventions [55, 56]. The ongoing malaria transmission in the study HDs was confirmed by high *Plasmodium* infection rates in children under five years-old using LLINs (30%), varying from one district to another, and among the clusters of the same HD. These data are consistent with those reported from a nationwide survey conducted after the LLIN distribution campaign in 2011 [57]. The above-mentioned study revealed 60% LLIN utilization, 36% malaria prevalence (using RDTs) in children less than six years-old sleeping under LLINs and 66% prevalence in children not using LLINs. Furthermore, poor housing quality, weaknesses in LLIN coverage (more than two people/net) and low-level education of the heads of the households were identified as the key factors leading to continuous malaria transmission despite LLIN interventions in Cameroon. Such socio-economic and cultural factors are preponderant in most households of the study HDs in North Cameroon, especially in Pitoa where malaria infection rates had increased (47%), compared with 34% recorded in 2002 by Etang et al. [58] in children not using ITNs. In addition, the present study underlined that high vector densities, availability of alternative hosts and deltamethrin resistance, worsened by the plasticity in mosquito feeding habits, are key entomological factors limiting the effectiveness of LLINs. The combination of socio-economic and entomological factors among others could therefore explain the increasing malaria infections in the three study HDs. Treatment of livestock with a long-lasting ivermectin formulation, as suggested by Chaccour et al. [59] in Kenya, may be trialled as a complementary tool for malaria vector control in addition to LLINs in North Cameroon.

## Conclusions

The present study highlights the behavioural plasticity of the major malaria vectors and continuous *Plasmodium* infection in the presence of LLINs. The high densities of the major vectors, species diversity and ability to feed on domestic animal as alternative hosts are key entomological factors increasing the complexity of vector control *via* LLINs in North Cameroon. Hence, complementary control measures are needed to sustain malaria control in North Cameroon.

## Data Availability

The data that support the findings of this study are available from the World Health Organization (WHO), but restrictions apply to the availability of these data, which were used under license for the present study and not publicly available. Data are, however, available from the authors upon reasonable request and with the permission of the WHO.

## Abbreviations

ANOVA: analysis of variance
CSP: circumsporozoite protein
DNA: deoxyribonucleic acid
ELISA: enzyme-linked immunosorbent assay
HBI: human blood index
HD: health districts
IRS: insecticide residual spraying
LLINs: long-lasting insecticidal nets
MINSANTE: Ministère de la Santé
OCEAC: Organisation de Coordination pour la lutte contre les Endémies en Afrique Centrale
OCP: outdoor clay-pots
PCR-RFLP: polymerase chain reaction-restriction fragment length polymorphism
PSC: pyrethrum spray catches
RDT: rapid diagnostic test
SSA: sub-Saharan Africa
WET: window exit traps
WHO: World Health Organization
